# Study on the onset mechanism of bio-blister degradation of polyolefin by diatom attachment in seawater

**DOI:** 10.1038/s41598-024-54668-8

**Published:** 2024-02-16

**Authors:** Hisayuki Nakatani, Shun Narizumi, Seiya Okubo, Suguru Motokucho, Anh Thi Ngoc Dao, Hee-Jin Kim, Mitsuharu Yagi, Yusaku Kyozuka, Shigenobu Miura, Kanth V. Josyula

**Affiliations:** 1 Polymeri Materials Laboratory, Chemistry and Materials Engineering Program, Nagasaki University, 1-14 Bunkyo-machi, Nagasaki, 852-8521 Japan; 2 Graduate School of Fisheries and Environmental Sciences, Nagasaki University, 1-14 Bunkyo-machi, Nagasaki, 852-8521 Japan; 3 Organization for Marine Science and Technology, Nagasaki University, 1-14 Bunkyo-machiachi, Nagasaki, 852-8521 Japan; 4 BioLogiQ Japan LLC, 3-9-10-347, Takaidohigashi, Suginami, Tokyo, 1680072 Japan; 5 BioLogiQ, Inc., 3834 Professional Way, Idaho Falls, ID 83402 USA

**Keywords:** Sustainability, Materials science, Environmental chemistry

## Abstract

It is essential to develop a mechanism for lowering the molecular weight of polyolefins to achieve biodegradation in seawater. In this study, a polypropylene/polylactic acid blend sample was first subjected to photodegradation pretreatment, and it was confirmed that in pure water, the acid generated promotes the polypropylene degradation (autoxidation), while in alkaline seawater, the promotion was inhibited by a neutralization reaction. In the autoxidation of polyolefins in alkaline seawater, aqueous Cl^−^ was also the inhibitor. However, we found that autoxidation could be initiated even in seawater by lowering the pH and using dissociation of ClOH (called blister degradation). The blister degradation mechanism enabled autoxidation, even in seawater, by taking advantage of the ability of diatoms to secrete transparent exopolymer particles (TEP) to prevent direct contact between the surface layer of polyolefins and alkaline seawater. We named blister degradation in seawater with diatoms as bio-blister degradation and confirmed its manifestation using linear low-density polyethylene (LLDPE)/starch samples by SEM, IR, DSC and GPC analysis.

## Introduction

Plastic litter can enter the sea from many sources and causes microplastic (MP) pollution^1–6^. Some MPs comprise polyolefins, such as polyethylene (PE) and polypropylene (PP), and are produced by degradation initiated by solar exposure^7–9^. A large molecule, such as a polyolefin, cannot easily pass into the cells of microorganisms and therefore, cannot be directly metabolized. However, polyolefins can be spontaneously degraded to low-molecular-weight products that are biodegradable^10,11^. We previously degraded PP in seawater via an advanced oxidation process (AOP)^12^. The seawater composition resulted in accelerated degradation process at a low pH^12^. The degradation of polyolefins caused by the dissociation of ClOH is called “blister deterioration” or “blister degradation”^13^ and can be induced in the sea by lowering the pH^12^. Seawater contains a wide variety of organic and inorganic compounds. Specifically, Cl^−^ reacts with some radical species in seawater to inhibit the initiation of autoxidation of plastics^14^. Thus, it is necessary to develop a mechanism to prevent direct contact between plastics and seawater. One useful method is the application of a biofilm to the surface of plastics wherein as soon as plastic litter drifts into the ocean, microalgae attach to and cover the surface of the plastic with a biofilm^15^. Delacuvellerie et al. reported the photodegradation of low-density polyethylene (LDPE) with an attached biofilm in a water column at a seawater depth of 4.5 m over 83 days of immersion in the Mediterranean Sea^15^. The biofilm caused the progressive degradation of LDPE in the sea. Biofilm formation prevents permanent contact with seawater and blocks the inhibition of autoxidation initiation by Cl^−^. Diatoms secrete acidic polysaccharides^16^ and generate a biofilm. The pH of the surface of the plastic litter to which the biofilm is attached is thereby lowered, and autoxidation is promoted.

In this study, the acceleration of polyolefin autoxidation induced by an acid-generation system was investigated for the photodegradation of a PP/polylactic acid (PLA) blend in pure water. It is well known that TiO_2_ works as a photocatalyst for autoxidation. However, the photocatalytic potential of TiO_2_ is inefficient in using visible light source such as sunshine due to having no absorption region at wavelengths above 385 nm, the development of TiO_2_ with absorption in the longer wavelength (Vis-TiO_2_) was required to use visible light source. Shang et. al. reported that CuPc-TiO_2_ nanoparticle powder showed a high PS photodegradation activity under visible light irradiation^17^. The PP/PLA photodegradation was performed using the same photocatalyst and adhesion of diatoms to the blend in seawater was then examined. Based on the study results, we performed bio-blister degradation of a linear low-density polyethylene (LLDPE)/starch blend under simulated sea conditions at the East Idaho Aquarium. The blend was suitable for observing the adhesion of diatoms to the blend and the matrix surface morphology. Bio-blister degradation was characterized by performing SEM/EDX, IR, DSC and GPC measurements.

## Materials and methods

### Materials

PP was supplied by Prime Polymer Co., Ltd. (product name: J-700GP). The weight-average molecular weight (Mw) and dispersity (Mw/Mn) were 2.9 × 10^5^ g mol^−1^ and 5.7. Polylactic acid (PLA) was supplied by Mitsui Chemicals, Inc. (production name: Gread H-100). The weight-average molecular weight (Mw) and dispersity (Mw/Mn) were 1.2 × 10^5^ g mol^−1^ and 1.1, respectively. Potassium persulfate (K_2_S_2_O_8_) and glycerin were purchased from Wako Pure Chemical Industries. Seawater was prepared with Gex artificial saltwater purchased from Amazon.co.jp. Copper phthalocyanine (CuPc) was purchased from Sigma-Aldrich Co. LLC. Bis(1-undecanoxy-2,2,6,6-tetramethylpiperidin-4-yl)carbonate (LA-81) was supplied by ADEKA Co. and was employed as hindered amine light stabilizer (HALS).

### Preparations of CuPc-modified TiO_2_ (CuPc-TiO_2_)

CuPc-TiO_2_ was prepared as a visible light absorbing photocatalyst with reference to Shang et al. paper^17^. 0.2 g-TiO_2_ was put into 50 ml-ethanol and then was vigorously stirred at r.t. The suspension was impregnated with 100 ml CuPc-ethanol solution (2.4 × 10^–4^ mol/L-CuPc) at 60 °C for 3 h and was washed with water for three times by centrifugalization. The precipitate was dried at 80 °C. The CuPc content was ca. 1.2% in the TiO_2_.

### Photodegradation pretreatment and biochemical oxygen demand (BOD) measurement method

Initially, PP and PLA (PP/PLA = 8/2 weight ratio) powders were prepared to a particle size of 0.30 mm or less using a freeze grinder. Then, the CuPc-TiO_2_ was prepared as an ethanol slurry solution and applied to PP and PLA. In addition, 0.14 phr of HALS was applied to PLA alone, and 20 phr of glycerin was also applied in the same manner. The PP and PLA were mixed, and the film was compress molded in a press at 200 °C for 10 min. A light-emitting diode (LED) lamp of 25 W (Yazawa Corporation Co., Ltd., CLED10012WH) was used as a visible light irradiation source. The distance between specimens and the lamp was ca. 40 cm, and its photo flux density was ca. 50 µmol/m^2^ s. After the visible light irradiation treatment (photodegradation) on the film was performed using for 24 h, the respirometric biodegradation test was carried out with BOD method using a TAITEC BOD tester (TAITEC Co., Ltd.,). The tester was composed of a 500 ml incubator bottle equipped with a pressure sensor and a magnetic stirrer. The tester system is based on a pressure measurement in a closed system: microorganisms in the sample consume oxygen and form CO_2_. The CO_2_ is absorbed by KOH, creating a vacuum which can be read directly as a measured value in mg/l BOD. The biodegradation test using natural soil containing fungi and other natural microorganisms was performed at 20 °C under the following conditions: The tester contained 400 ml deionized water, the sample (20 mm × 5 mm × 0.05 mm), 400 mg soil picked around Nagasaki university, Japan and salt solutions having K_2_HPO_4_ 34.0 mg, KH_2_PO_4_ 87.2 mg, Na_2_HPO_4_ 13.4 mg, MgSO_4_·7H_2_O 9.2 mg, FeCl_3_·6H_2_O 0.2 mg, CaCl_2_·2H_2_O 14.4 mg and NH_4_NO_3_ 4.0 mg. The BOD value was measured under stirring (ca. 200 rpm). The biodegradation extent (mineralization percentage) of each treated sample was obtained from as follows: [(carbon amount of CO_2_ generated from sample) − (carbon amount of unbiodegraded sample)]/(all carbon amount of sample).

### Bio-blister degradation test at East Idaho Aquarium

A bio-blister degradation test was performed at the East Idaho Aquarium (570 E Anderson St, Idaho Falls, ID 8,401, USA) using a linear low density polyethylene (LLDPE: 60%)/starch with a plasticizer (NPQ: 40%) blow molded film produced by BioLogiQ, Inc.. The characteristic information of LLDPE (ExxonMobil Enable 20-10ME) part was as follows: Density = 0.920 g/cm^3^, melt index (190 °C/2.16 kg) = 1.0 g/10 min peak and melting temperature = 114 °C. The NPQ was mainly composed of starch and was made by mixing with a small amount of plasticizer such as glycerin (Japanese Patent No. 6949736 and 6943772). 0.010 mm thick films of the LLDPE and LLDPE/NPQ were used for the bio-blister degradation test. These films were cut to a size of 114 mm × 64 mm, were surrounded with a wire mesh and were placed between metal frames in water tanks (see Figure S1). The installation conditions in each water tank were as follows. Shark tank: depth 3.35 m, seawater temp = 25.7 °C, salinity = 1.019 specific gravity, pH = 7.9. Amazon (River giants): depth 1.52 m, water temp, = 22.7 °C, salinity = 1.0 specific gravity, pH = 7.3.

### Fourier transform infrared (FT-IR) analysis

The IR spectra 16 scans were measured with an FT-IR spectrometer (Jasco FT-IR 660 plus) at a resolution of 4 cm^−1^ over the full mid-IR range (400–4000 cm^−1^).

### Scanning electron microscope (SEM) with energy dispersive X-ray spectroscopy analysis

The SEM / EDX analysis was carried out with a JSM-7500FAM (JEOL) with 5.0 kV. The working distance was about 3 × 4 mm. Samples were placed in dried oven maintained at 27 °C for 30 min and were sputter-coated with gold before SEM imaging.

### Microscope observation

The microscope observation was carried out a digital microscope (Keyence VHX-8000, Japan).

### Seawater sampling method

Seawater sampling was carried out in the sea at 32° 46ʹ 49ʺ N and 129° 43ʹ 23ʺ E near Nagasaki-city, Japan. The retrieving was performed with a training vessel T/V Kakuyo-maru (155 gross tonnage: Faculty of Fisheries, Nagasaki University)^18,19^.

### Differential scanning calorimetry (DSC) measurement

DSC measurements were made with a SHIMADZU DSC-60 Plus. The samples of about 5 mg weight were sealed in aluminum pans. The measurement of the samples was carried out at a heating rate of 10 °C/min under a nitrogen atmosphere. The data was taken on the 1st run.

### Gel permeation chromatography (GPC) analysis

LLDPE/NPQ samples were dissolved in 1, 2, 4-trichlorobenzene and were measured at a concentration of 0.5 mg/ml. Since small insoluble substances were observed in the sample solution, they were removed by heat filtration using a sintered filter with a pore diameter of 0.5 μm, and only the soluble portion was subjected to GPC measurement. The molecular weights were directly measured by HLC-8321GPC/HT GPC system (Tosoh Co., Ltd.) at 140 °C and determined by a weight average molecular weight in terms of polyethylene.

### Blister degradation using advance oxidation process (AOP)

To prove that the bio-blister degradation in the shark tank was the same reaction as (chemical) blister degradation, we performed chemical blister degradation on identical LLDPE/NPQ films in the seawater. The blister degradation was employed with a AOP using K_2_S_2_O_8_ seawater solution. The 15 days blister degradation procedure of the LLDPE/NPQ sample was according to reports^12^. (1) Five pieces of each film were put into a 100 ml glass vessel containing with a 20 ml of seawater solution with 0.54 g K_2_S_2_O_8_ at 65 °C for 12 h under stirring with a stirrer tip speed of ca. 100 rpm. (2) The equal amount of K_2_S_2_O_8_ seawater solution was added to compensate for the consumption of oxidant, and its degradation was carried out for 12 h under the same conditions. (3) The five pieces of the film were then transferred to a new 100 ml glass vessel containing 20 ml of seawater solution with 0.54 g K_2_S_2_O_8_, and the 15 days blister degradation was restarted under the same condition and was carried out for 15 days using (1)–(3) as one set. The pH value of solution was changed from 8.2 to 3 during each set.

## Results and discussion

### Influence of pH value on the progress of autoxidation of PP

**Figure 1:**
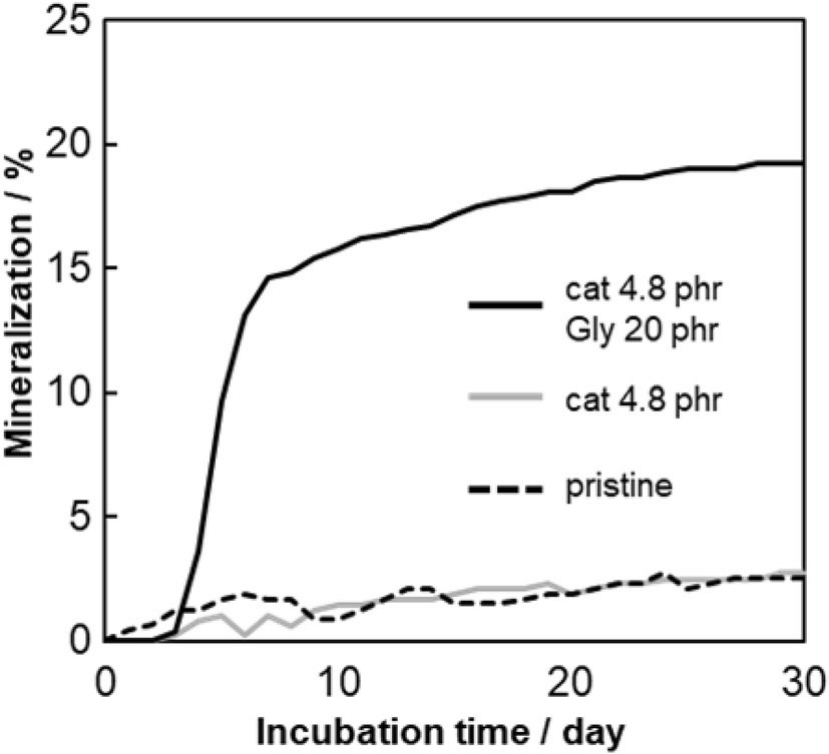
Mineralization versus incubation time of three kinds of PP/PLA/HALS blend samples in pure water: PP/PLA weight ratio = 8/2. HALS cont. = 0.14 phr. cat = CuPc-TiO_2_ catalyst. Gly = glycerin.

**Figure 2:**
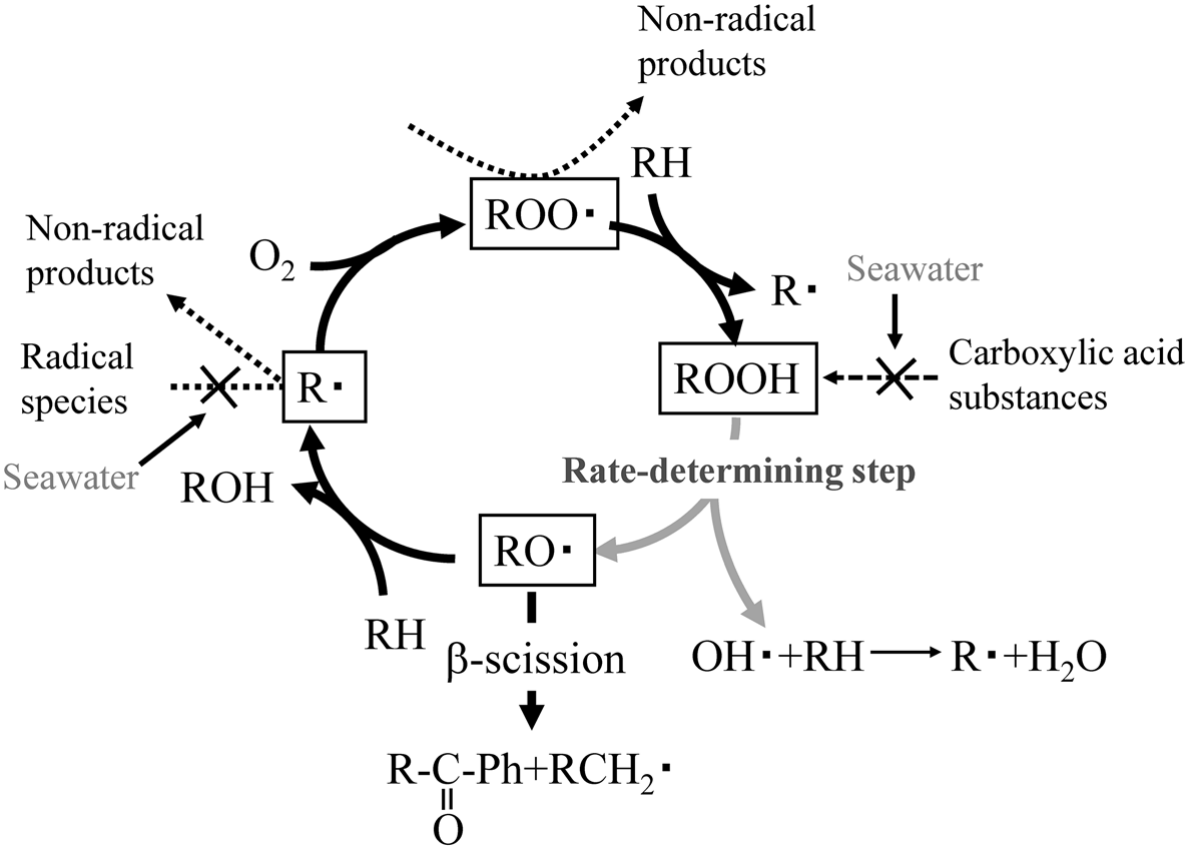
Autoxidation inhibition mechanism by seawater.

A pre-photodegradation treatment is used to generate hydroperoxide groups in the PP component of the blend sample. Autoxidation is initiated at these groups, generating carboxylic acid substances that promote autoxidation of the PLA component of the blend. Figure 1 shows the change in the mineralization values of PP/PLA/HALS blend samples determined from BOD measurements made in pure water. The blend sample containing 4.8 phr CuPc–TiO_2_ catalyst and 20 phr glycerin has a ca. 20% mineralization rate, i.e., biodegradation rate. This sample exhibits a considerably higher biodegradation rate than those of samples without added glycerin. The biodegradation rate of glycerin alone was approximately 1.9%, clearly indicating that the biodegrability of the blend sample itself was improved. The additive effect shown by glycerin is to improve the hydrophilicity of the PP component. In addition, Glycerin, a low molecular weight substance, has excellent mobility and serves as a medium for transporting macro radicals, peroxides, and organic acids generated by the autoxidation, diffusing the degradation throughout the PP matrix. Furthermore, the drastic improvement in the biodegradation of the sample results is due to the decomposition products of PLA, i.e., carboxylic acid substances, acting as an accelerator of autoxidation^20^. That is, autoxidation of the PLA component produces anhydride functional groups, which in turn cause the formation of carbonyl acid compounds that accelerate autoxidation^21^. This result implies that the generation of acidic substances during the autoxidation would cause the PP chain to be cleaved in water. By comparison, a similar improvement in the biodegradability of blend samples is not observed in the BOD test when seawater is used instead of water. The alkalinity of the seawater causes extreme lowering of the biodegradation performance of the blend sample. This result is obtained because alkaline seawater neutralizes the acid compounds produced by PLA degradation. Figure 2 shows that the aforementioned acid compounds are neutralized in alkaline seawater and an acceleration effect on autoxidation is not observed.

Figure 2 also shows that autoxidation is unlikely to occur in seawater, even at the initiation stage. Salinity lowers the degree of autoxidation of polyolefins because aqueous Cl^−^ inhibits the production of alkyl radicals in seawater^14^. The suppression mechanism is as follows:

1 $$ {\text{Cl}}^{ - } + {\text{OH}}^{ \cdot } \to {\text{ClOH}}^{ \cdot - } \rightleftarrows {\text{Cl}}^{ \cdot } + {\text{OH}}^{ - } $$

2 $$ {\text{2Cl}}^{ \cdot } \to {\text{Cl}}_{{2}} $$

3 $$ {\text{Cl}}_{{2}} + {\text{2H}}_{{2}} {\text{O}} \to {\text{ClOH}} + {\text{Cl}}^{ - } + {\text{H}}_{{3}} {\text{O}}^{ + } $$

4 $$ {\text{ClOH}} \rightleftarrows {\text{Cl}}^{ \cdot } + {\text{OH}}^{ \cdot } $$

5 $$ {\text{ClOH}} + {\text{2H}}_{{2}} {\text{O}} \rightleftarrows {\text{ClO}}^{ - } + {\text{H}}_{{3}} {\text{O}}^{ + } $$

The inhibitory reaction is caused by Reaction (1) between Cl^−^ and radical species, i.e., OH^·^, generated by solar exposure to form Cl^·^. Two Cl^·^ atoms couple to produce Cl_2_, which then reacts with H_2_O to form ClOH [Reactions (2)–(5)]. The equilibria of the two aforementioned reactions for the formation and coupling of Cl^·^ atoms depends on the pH of the reaction medium^22^. As seawater has a pH of ca. 8.2, the reaction equilibrium (5) is shifted toward ClO^−^, which is less reactive than ClOH, suppressing the autoxidation of polyolefins in seawater. The degradation of polyolefins caused by the dissociation of ClOH is called “blister degradation” and can be induced in the sea by lowering the pH^12^. Thus, to spontaneously initiate the blister degradation of polyolefins in seawater, direct contact between seawater and the polyolefin surface must be prevented.

### Bio-blister degradation of polyolefins in seawater using biofilm formation by diatom

**Figure 3:**
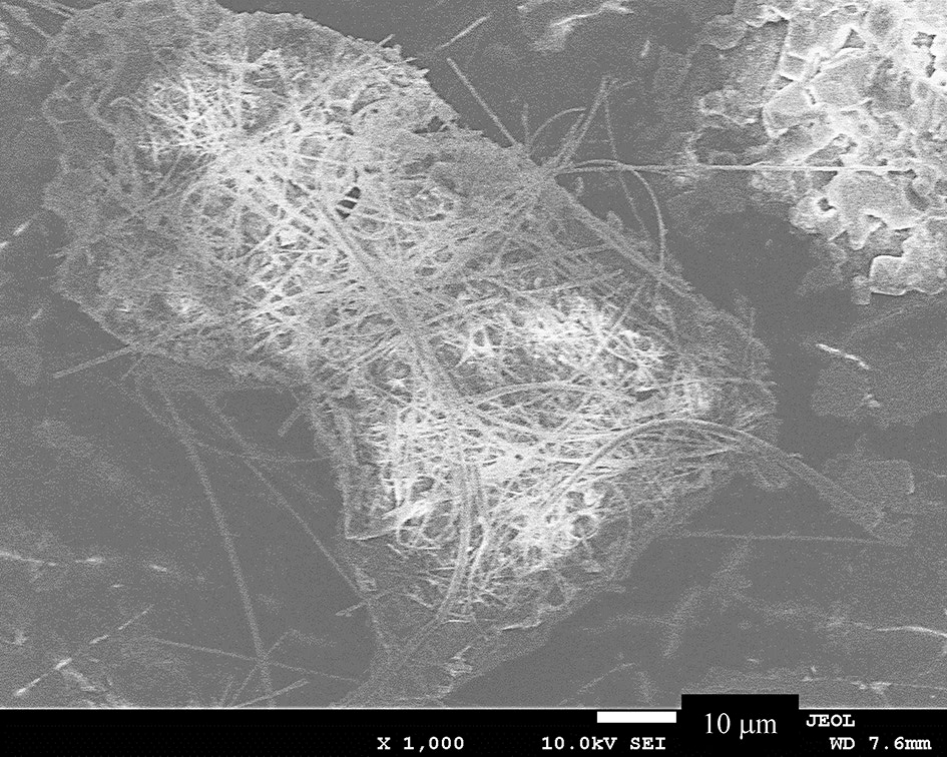
SEM image of pretreated degraded PP added soluble Si in the seawater: Incubation temperature 20 °C. Culture time = 28 days.

**Figure 4:**
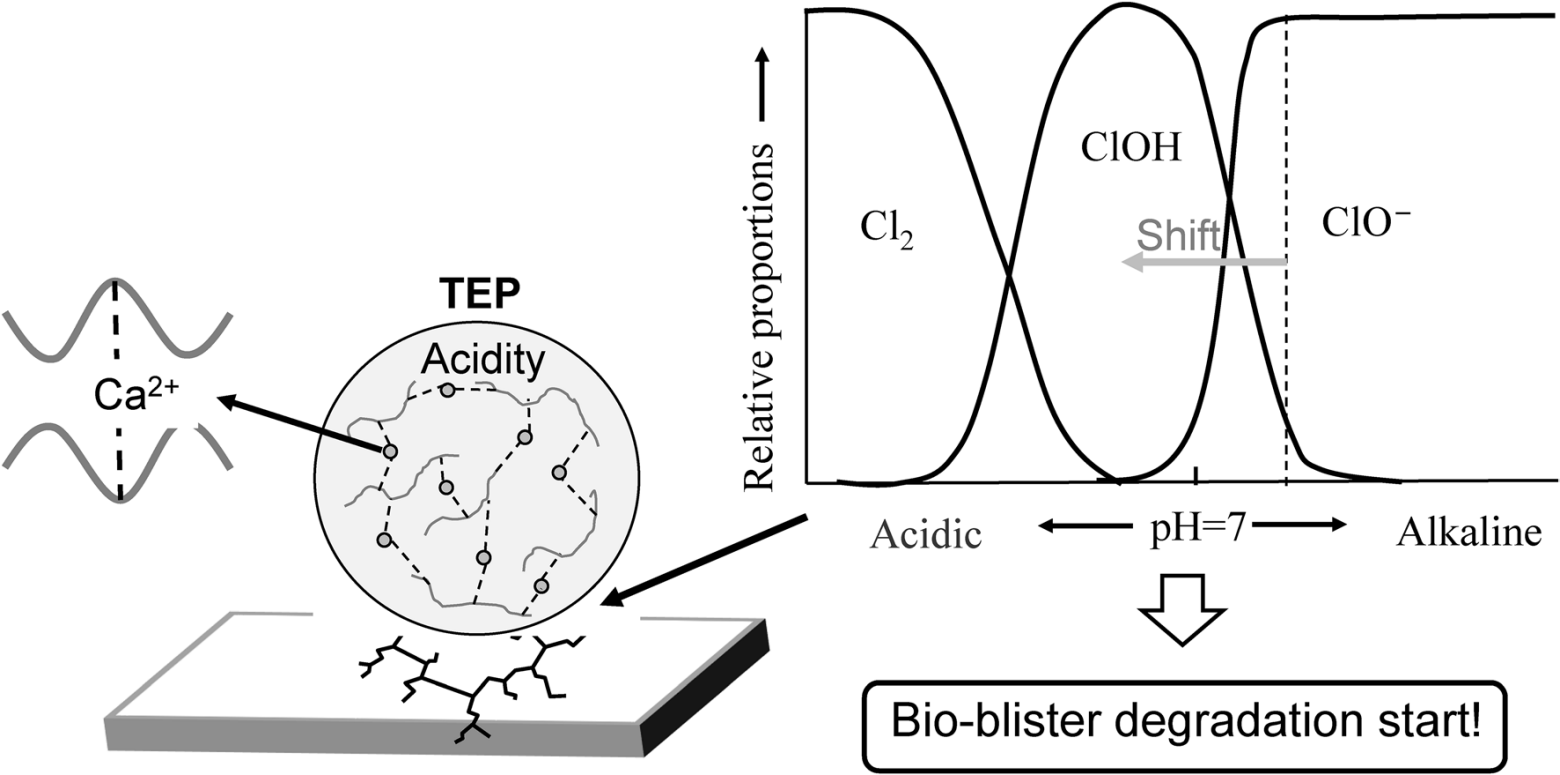
Bio-blister degradation with an equilibrium transfer induced by contact with acidic TEP.

Figure 3 shows an SEM image of degraded PP pretreated by adding soluble Si to seawater. Microalgae (mainly diatoms) form a dense biofilm on the surface of the pretreated degraded PP. This result indicates that a biofilm can be formed on the surface of a polyolefin, such as PP, in a short time by improving the surface hydrophilicity. Akagi et al. reported that diatoms secreted transparent exopolymer particles (TEP) to dissolve feldspars, which were subsequently incorporated into the diatom cells as frustule material^23^. The authors claimed that diatoms produce TEP to assimilate silicic acid from dusts^23^, which are a residual source of silica in the environment. After a diatom bloom, diatom cells do not contain sufficient silicon; thus, TEP (an acidic polysaccharide gel^16,24^) is secreted to the diatom body surface, and silicon is taken into the body by acid dissolution for use as a frustule raw material^23^. The acidic gel is cross-linked with calcium ions (Ca^2+^)^23^ and does not diffuse in seawater. Thus, creating conditions for diatoms stick to the surface of a polyolefin, i.e., PP or PE, and discharge TEP could acidify the polyolefin surface, even in seawater. In our previous study^12^, the pH of seawater was decreased by the addition of a K_2_S_2_O_8_ initiator. The autoxidation of PP proceeded uniformly regardless of depth, and the MP formation rate was significantly accelerated^12^. Figure 4 shows the occurrence of a new degradation process, i.e., bio-blister degradation, in which the equilibrium transfer of ClO^−^ to ClOH was induced by contact between the polyolefin surface and acidic TEP cross-linked with Ca^2+^ on the diatom surface. The ClOH species has a longer lifetime than OH^·^^22^. The ClOH species migrates deeply into the polymer matrix before dissociating into radicals and initiating autoxidation^14^. Additionally, the acidic TEP acts as a catalyst to decompose hydroperoxide, accelerating the progress of autoxidation. The adhesion of acidic TEP is considered to degrade polyolefins in the sea, even in the absence of sunlight irradiation. We named this degradation of polyolefins by diatoms “bio-blister degradation”.

Biofilm formation occurs differently on PP and PE, which are representative polyolefins. Lagarde et al. reported different types of long-term colonization for PP and PE^25^. It is likely that a biofilm forms on PP by the creation of heteroaggregates that adhere to PP in fine pieces, making it very difficult to observe changes in the PP surface. This aggregation process is not observed during biofilm formation on PE, making it easier to observe the surface on which the biofilm forms. PE is suitable for confirming that bio-blister degradation of polyolefin is caused by diatoms in seawater. In this study, the bio-blister degradation process was observed for PE (LLDPE and LLDPE/starch with a plasticizer: NPQ) films in tanks of seawater (a shark tank) and freshwater (Amazon River Giants) in the East Idaho Aquarium, USA. We chose LLDPE because of its adjustable flexibility and stiffness as a PE film. Because other PEs, i.e., high-density PE and low-density PE, were either too stiff or too soft as a film and were not suitable for long-term suspension in the aquarium.

**Figure 5:**
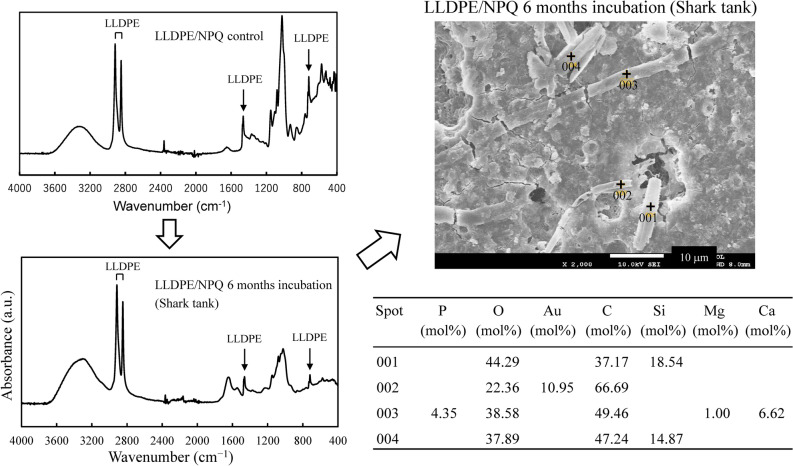
IR spectrum of LLDPE/NPQ control film, and its IR spectrum change, SEM image and EDX data after 6 months bio-blister degradation test in Shark tank at East Idaho Aquarium.

**Figure 6:**
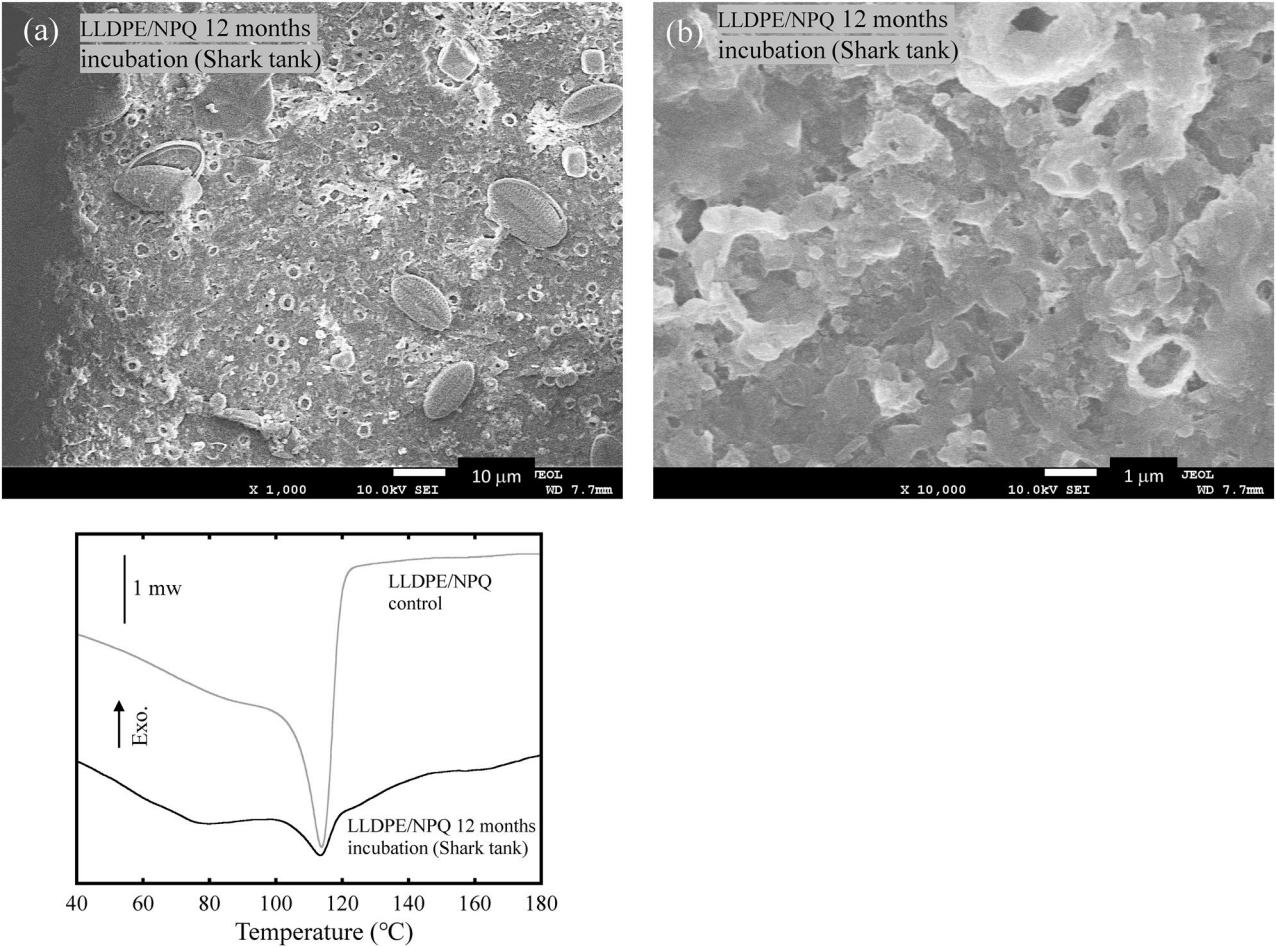
SEM images of LLDPE/NPQ sample after 12 months bio-blister degradation test in shark tank at East Idaho Aquarium: (**a**) 1000 and (**b**) 10,000 times magnifications, respectively, and comparison of DSC curves between control and 12 months incubation samples.

Figure S1 shows a considerable difference in the appearance of the commercial LLDPE and LLDPE/NPQ film samples in seawater after 12 months of bio-blister degradation. Large area defects are found in the LLDPE/NPQ film, whereas the shape of the commercial LLDPE is maintained. Figure S2 shows microphotographs of the LLDPE/NPQ films after a six-month-long bio-blister degradation test. A hole defect can be observed in an LLDPE/NPQ film, where the edge of the hole is not sharp but stringy, suggesting that the defect was formed by the LLDPE dissolution. In addition, a pennate diatom can be observed on the film surface (the white arrow in Figure S2). Figure 5 shows the IR spectrum of a LLDPE/NPQ control sample. The peaks at 2917, 2843, 1464, and 720 cm^–1^ correspond to LLDPE^26^. Relatively broad peaks around 3400, 1650, and 1110 cm^–1^ can be observed in addition to peaks derived from the LLDPE component of the sample. The positions of these broad peaks coincide with those of polysaccharides^27^, which are likely derived from diatoms and other microalgae present on the sample surface. Figure 5 shows that the peaks in the FT–IR spectrum of the LLDPE/NPQ sample after the six-month-long bio-blister degradation test in the shark tank occur in the same positions as for that of the control sample. An algal-generated biofilm, such as TEP, is present on the film surface. As this biofilm consists of a type of polysaccharide, the peaks in the IR spectrum of the biofilm appear at the same position as in the IR spectrum of starch. However, the intensities of the individual peaks differ between the IR spectra of the biofilm and starch. Compared to the IR spectrum of starch, in the IR spectrum of the biofilm, the intensities of the peaks around 3400 and 1650 cm^–1^ are higher and the intensity of the peak around 1110 cm^–1^ is considerably lower. This result indicates that the starch component is selectively replaced by the biofilm. The SEM/EDX analysis shows there are two types of algae on the film surface. In the EDX analysis, elemental Si is detected in the alga marked 001 and 004, which is therefore categorized as a diatom, whereas elemental Ca is detected in the alga marked 003, which is therefore categorized as a blue-green alga. Elemental Ca is not detected in the alga marked 002, which, however, has the same cylindrical shape as that of 003 and is therefore likely to be the same blue-green alga. Figure S3 shows that LLDPE/NPQ is a polymer blend with a sea–island structure, in which the islands composed of starch-based NPQ range from 100 nm to 1.5 µm in size. A 10 µm diameter pit can be observed around the diatom marked 001 in Figure 5. The pit is considerably larger than the NPQ domain, indicating that the LLDPE matrix has been degraded in addition to NPQ. Biodegradation of PE is generally extremely slow. However, the degradability (including the biodegradability) of PE can be improved by blending PE with starch^27,29,30^. This improvement results from an increase in the surface area of the PE matrix because the porosity of PE is increased by first biodegrading and dissipating the easily biodegradable starch component^28,29^. The improved biodegradability of LLDPE/NPQ also results from selective biodegradation of the NPQ domains, which are nanosized and dispersed in the PE matrix. Figure 6 shows SEM images of the LLDPE/NPQ sample obtained after a 12-month long bio-blister degradation test in the shark tank at the East Idaho Aquarium. In Fig. 6(a), multiple pennate diatoms with long diameters exceeding 10 µm can be observed on the rough sample surface. Figure S4 is a magnified SEM image of some of these pennate diatoms. Numerous nanosized particles can be observed on the diatom frustules. These nanosized particles are considered to consist of the degraded LLDPE component. In the corresponding magnified SEM image shown in Fig. 6(b), the sample surface is deeply eroded. In the DSC curves shown in Fig. 6, the intensity of the melting-point peak corresponding to the LLDPE component is considerably lower than that of the control sample. The melting peak of the incubated sample is broader and shorter than that of the control sample. These differences between the results for the control and incubated samples can be attributed to diatom-induced bio-blister degradation over 12 months in the shark tank. Unlike the surface SEM images of the samples incubated for six months, the surface SEM images of the samples incubated for 12 months indicate uniform erosion over the entire surface.

Figure S5 shows the EDX data and SEM images of the LLDPE/NPQ sample after the 12-month long bio-blister degradation test in the freshwater (Amazon River Giants) tank at the East Idaho Aquarium. Diatoms with similar sizes to those observed for the samples incubated in the shark tank can be observed attached to the LLDPE/NPQ samples. However, the surface of the matrix is smooth with only a few cracks, which is in stark contrast to the eroded, uneven surface of the sample degraded in the shark tank. There appears to be little or no progress of bio-blister degradation in the freshwater tank. Figure S6 shows the conditions of the seawater and freshwater tanks and compares the DSC curves of the LLDPE/NPQ samples. As the freshwater (Amazon River Giants) has no salinity, i.e., no chloride ions are present, bio-blister degradation of the sample does not occur in the freshwater tank. The intensity of the DSC melting curve of the incubated sample is considerably lower than that of the control sample. The decrease in the peak intensity of the DSC curve of the incubated sample compared to that of the control sample mainly results from the apparent decrease in the percentage of the LLDPE component in the incubated sample caused by the adhesion of diatoms and other microorganisms to the sample surface during 12 months of incubation. The intensity of the melting peak in the DSC curve is considerably higher for the sample degraded over 12 months of incubation in freshwater than that of the sample degraded over 12 months of incubation in seawater. The peak in the DSC curve of the sample degraded in seawater indicates exothermic behavior above 120 °C, probably because of pyrolysis of the degraded LLDPE component. In addition, the baseline of 12 months of incubation in seawater is very fluctuating. This may be due to degradation of the LLDPE component and thermal decomposition of adhering diatoms. By contrast, a peak plateau is observed for the DSC curve of the freshwater sample, indicating little degradation.

**Figure 7:**
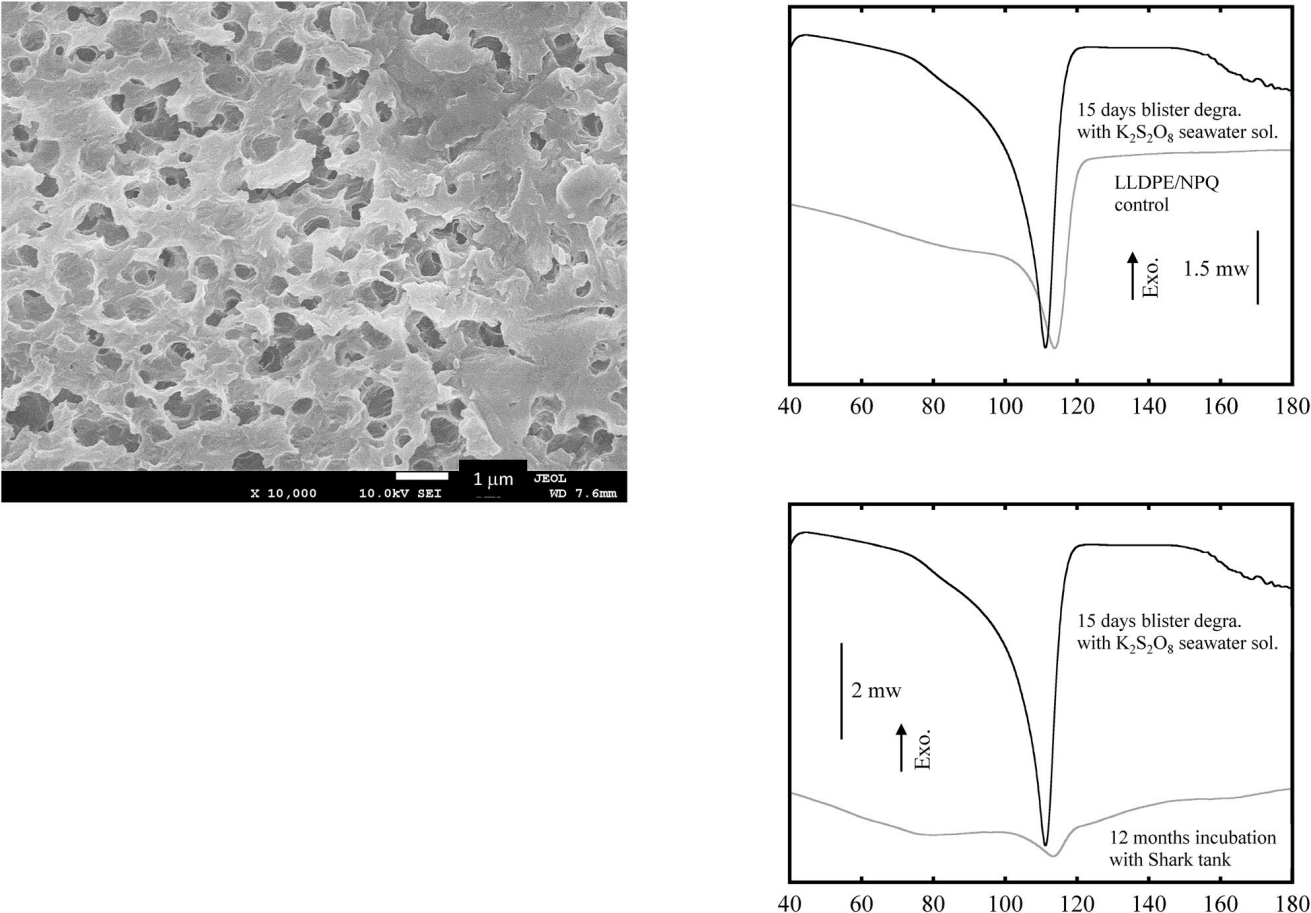
SEM images of LLDPE/NPQ sample after 15 days blister degradation test with K_2_S_2_O_8_ seawater solution, and comparison of DSC curves of control and 15 days blister degradation samples with K_2_S_2_O_8_ seawater solution or 12 months incubation sample with Shark tank.

**Figure 8:**
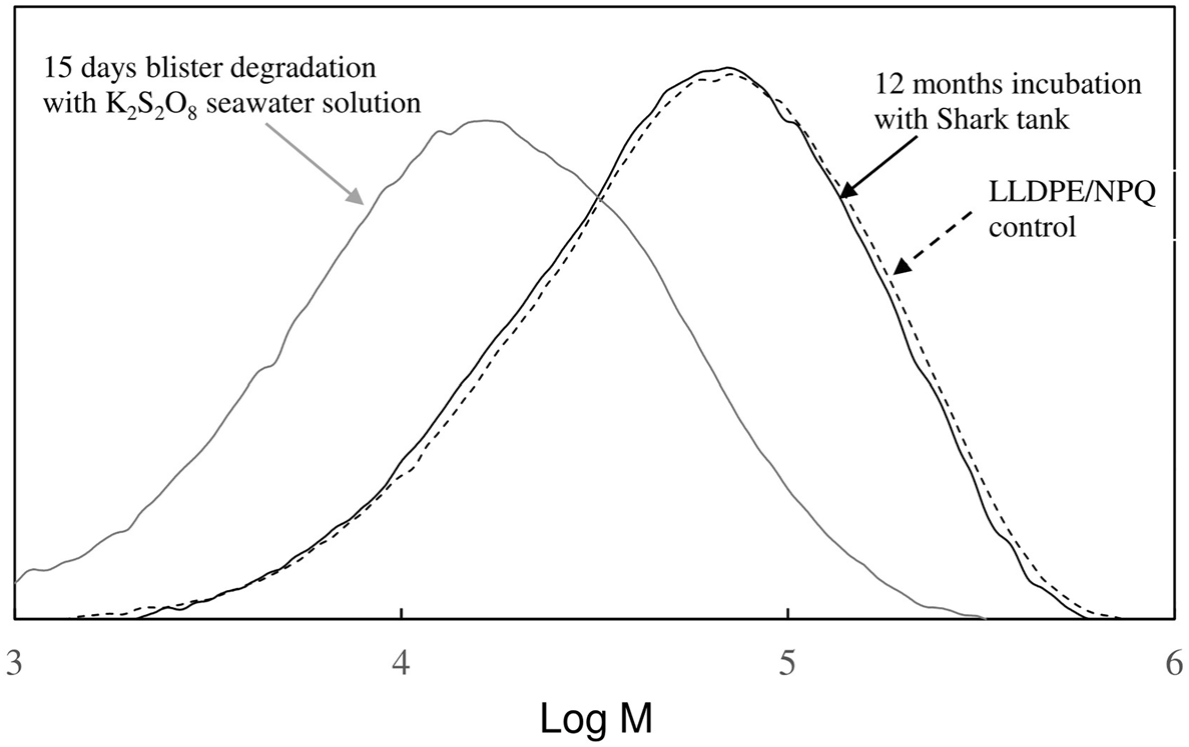
Molecular weight curves of LLDPE/NPQ samples: Control, 12 months incubation with Shark tank and 15 days blister degradation with K_2_S_2_O_8_ seawater solution.

Figure 7 shows SEM images of the LLDPE/NPQ sample after a 15-day blister degradation test based on the AOP method (using a K_2_S_2_O_8_ seawater solution) and compares the DSC curves of the control sample and the sample incubated for 12 months in the shark tank. The surface of the LLDPE/NPQ sample subjected to 15 days of blister degradation is as heavily eroded as the sample subjected to bio-blister degradation for 12 months in the shark tank. Numerous spherical pits with diameters of several hundred nanometers are observed in the SEM photograph of the15-day blister degradation using the AOP method, corresponding to traces of the NPQ component mainly composed of starch. Compared to the results for the control sample, the intensity of the melting peak in the DSC curve of the incubated sample is considerably higher and the corresponding fusion enthalpy is approximately three times larger. The high intensity of the melting peak in the DSC curve of the incubated sample is attributed to the LLDPE component because blister degradation selectively degrades nearly all the starch component and also breaks some LLDPE chains, causing chemical crystallization and a subsequent increase in the sample crystallinity^30^. The melting point of the incubated sample is approximately 3 °C lower than that of the control sample. This result also indicates that the LLDPE component has been severely degraded. Figure 7 shows that the intensity of the melting peak in the DSC curve of the sample subjected to bio-blister degradation in the shark tank is approximately 1/6th of that for the sample subjected to 15 days of blister degradation in the K_2_S_2_O_8_ seawater solution. This result is obtained because the sample incubated in the shark tank has a high percentage of the residual starch component and a relatively low percentage of the LLDPE component resulting from biofilm development. The melting point of the sample incubated in the shark tank is approximately 2 °C higher than that of the sample incubated in the seawater solution. The changes in the thermal properties of the samples with time show that the rate of bio-blister degradation is considerably lower than that of blister degradation using the K_2_S_2_O_8_ seawater solution. The DSC curve of the sample subjected to bio-blister degradation contains an additional broad melting peak around 80 °C. This melting point is close to that of linear unsaturated hydrocarbon compounds with approximately 20 carbons, such as arachidic acid. The compound with this melting peak has probably accumulated in the body of the diatom. The numerous nanosized particles observed on the surface of the diatoms on the sample subjected to bio-blister degradation would consist of this compound with 20 carbons immediately before the particles are taken into the body of the diatom (see Figure S4). Figure 8 shows the molecular weight curves of LLDPE/NPQ samples, and their molecular weight information is summarized in Table S1. The molecular weight of LLDPE in the 15 days blister degraded sample shows a sharp decrease, decreasing by 75% in Mn, 69% in Mw and 62% in Mz compared to those of the control it. On the other hand, the molecular weight curve for the bio-blister degraded (12 month incubation in shark tank) sample shifts much less toward the low molecular weight side as shown in Figure 8. The decreasing rate is 0% for Mn, 5% for Mw, and 6% for Mz (see Table S1). The bio-blister degradation has occurred is confirmed by the decrease in molecular weight, which is significantly less than the changes in surface morphology and melting curves of the sample described above. The small change in the molecular weight measurement in the bio-blister degradation may be due to the incorporation of the degraded low molecular weight part into the diatoms. The molecular weight measurement removed 1, 2, 4-trichlorobenzene-insoluble diatoms through the filter (see Materials and methods section), which simultaneously might do the low molecular weight component of LLDPE present inside as well. The small change in molecular weight is likely due to diatoms storing lower molecular weight LLDPE.

## Conclusion

A mechanism for rapidly lowering the molecular weight of a polymeric matrix is important for the biodegradation of polyolefins, such as PP and PE. In this study, a PP/PLA blend sample was subjected to pre-photodegradation treatment to generate hydroperoxide groups in the PP component of the sample for initiating autoxidation and carboxylic acid substances in the PLA component to promote autoxidation. Adding a CuPc–TiO_2_ catalyst and glycerin to the blend sample resulted in a ca. 20% biodegradation rate in pure water. However, the generated carboxylic acid substances are neutralized in seawater, which is alkaline, disabling the acid-activated autoxidation mechanism for developing biodegradability of the blend. Aqueous Cl^−^ was found to be an effective inhibitor of autoxidation of polyolefins in seawater. However, autoxidation could be initiated by the dissociation of ClOH (called blister degradation) to lower the pH of seawater. Bio-blister degradation mechanism was found to enable autoxidation, even in seawater, by taking advantage of the TEP-secreting properties of diatoms to prevent direct contact between the surface layer of the blend and seawater. The bio-blister degradation caused by diatoms was studied for an LLDPE/NPQ sample. Characterization of this sample was showed that preferential decomposition of the starch component during blister degradation increased the porosity and consequently, the surface area, of the sample. The bio-blister degradation was carried out on LLDPE/NPQ samples in seawater and freshwater tanks in an aquarium. The results of SEM, IR, and DSC analyses revealed diatom-induced disintegration of the LLDPE component of the blend for the seawater sample but not the freshwater sample. Compared to the sample subjected to blister degradation in K_2_S_2_O_8_ seawater solution for 15 days, the sample subjected to bio-blister degradation in the seawater tank of the aquarium for 12 months exhibited a lower degree of erosion of the LLDPE component. The results of a DSC analysis on the samples indicated that bio-blister degradation occurred at a considerably lower rate than blister degradation and involved the accumulation of a 20-carbon degradation product in the bodies of the diatoms attached to the sample. The possibility that diatoms store such low molecular weight components was suggested by the change in molecular weight measured by GPC as well.

### Supplementary Information

Supplementary Information.

## Data Availability

The data that support the findings of this study are available from the corresponding author, [HN], upon reasonable request.
