# Supplementary material for: Study on the onset mechanism of bio-blister degradation of polyolefin by diatom attachment in seawater
Source: Sci Rep. 2024 Feb 16;14:3902. doi: 10.1038/s41598-024-54668-8 (PMC10873352; doi:10.1038/s41598-024-54668-8)
Supplement: Supplementary file 1 — Supplementary Information. [file 41598_2024_54668_MOESM1_ESM.pdf]

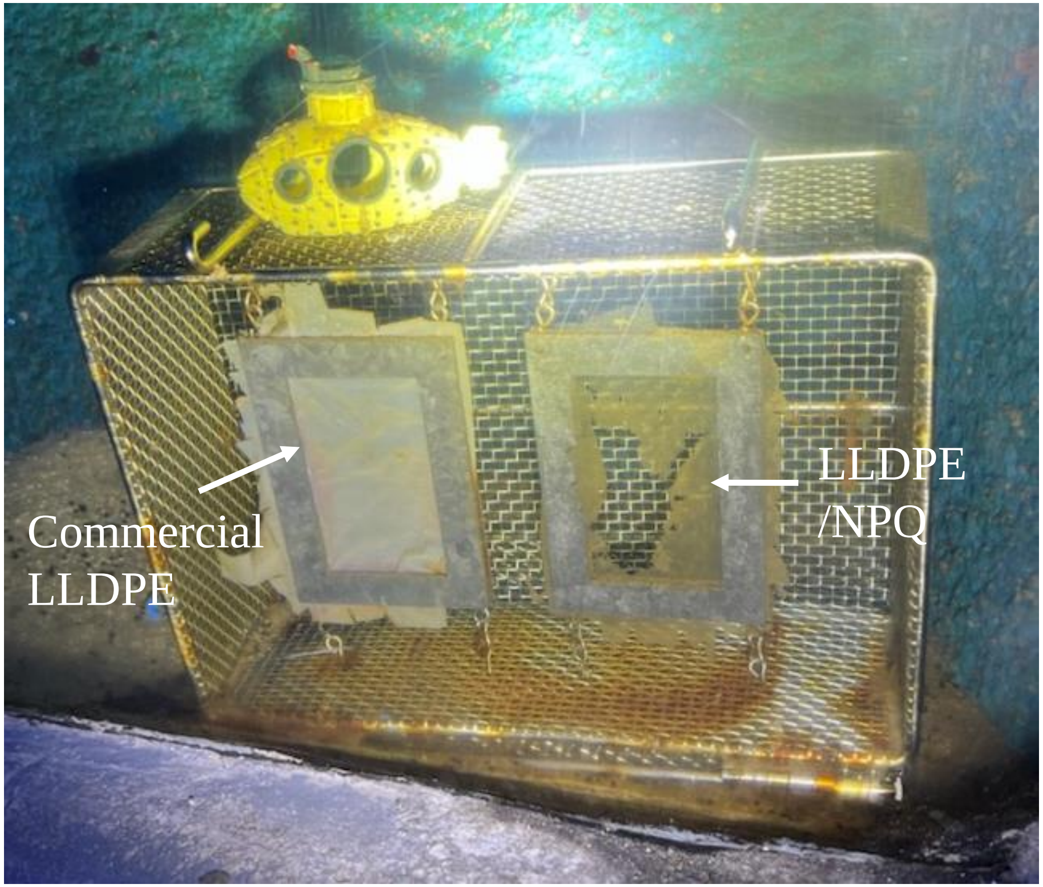

Figure S1 Bio-blister degradation test of commercial LLDPE and LLDPE/NPQ films in Shark tank at East Idaho Aquarium for 12 months.

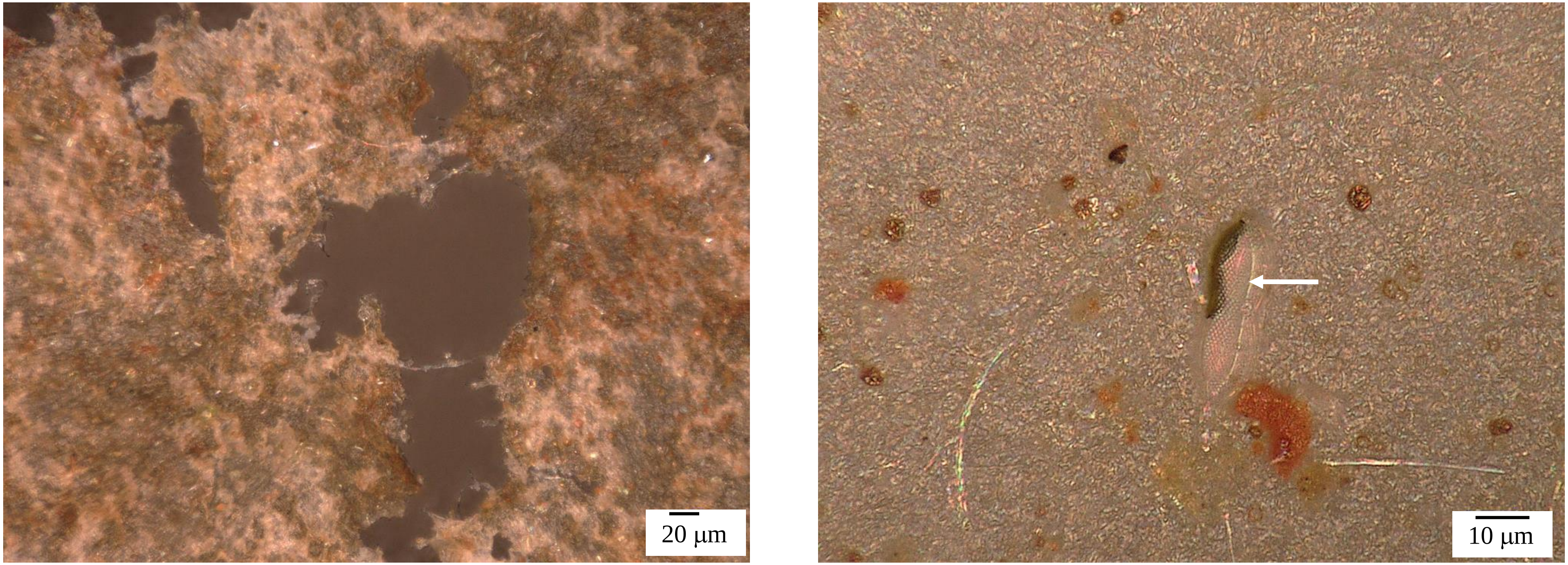

Figure S2 Microphotographs of LLDPE/NPQ films after bio-blister degradation test in shark tank at East Idaho Aquarium for 6 months.

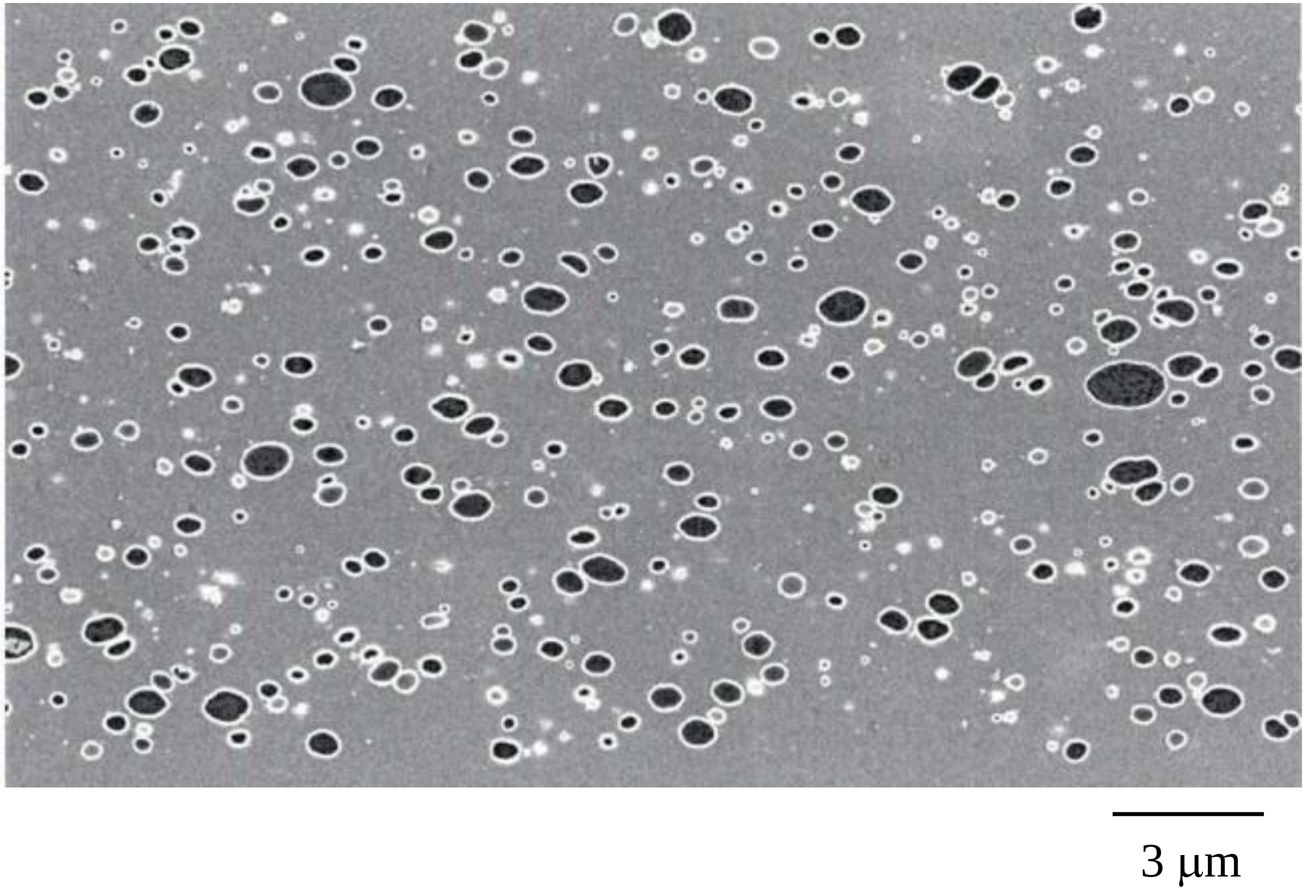

Figure S3 SEM image of LLDPE/NPQ control sample small piece cut with a diamond knife.

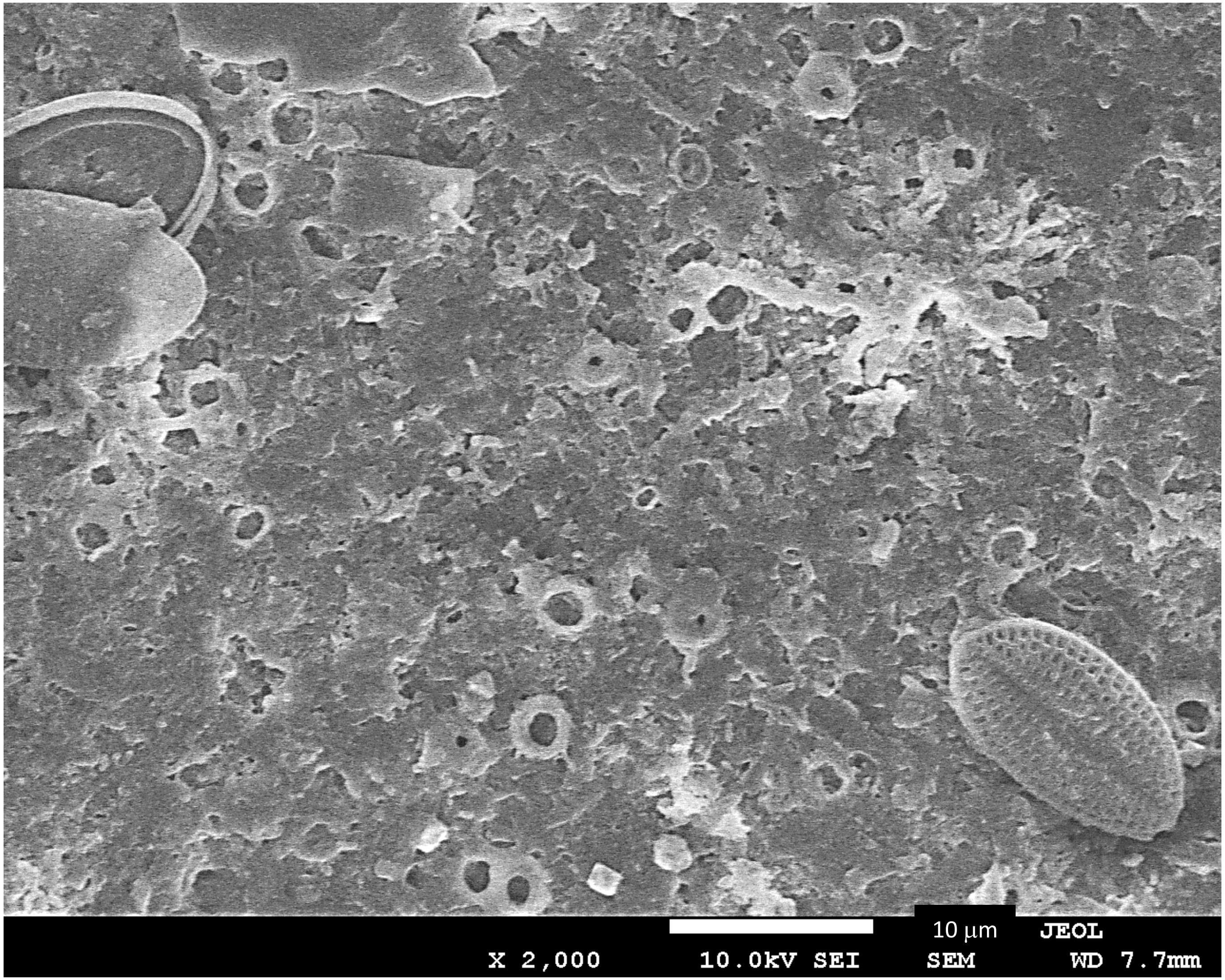

Figure S4 Magnified SEM image of LLDPE/NPQ sample after 12 months bio-blister degradation test in shark tank at East Idaho Aquarium.

Sample8_EDS-001

	O (mol%)	Au (mol%)	C (mol%)	Si (mol%)
001	41.16	13.21	25.61	20.03
002	44.73	14.48	19.06	21.72
003	36.09	12.20	35.44	16.28
004	38.38	10.63	36.38	14.60

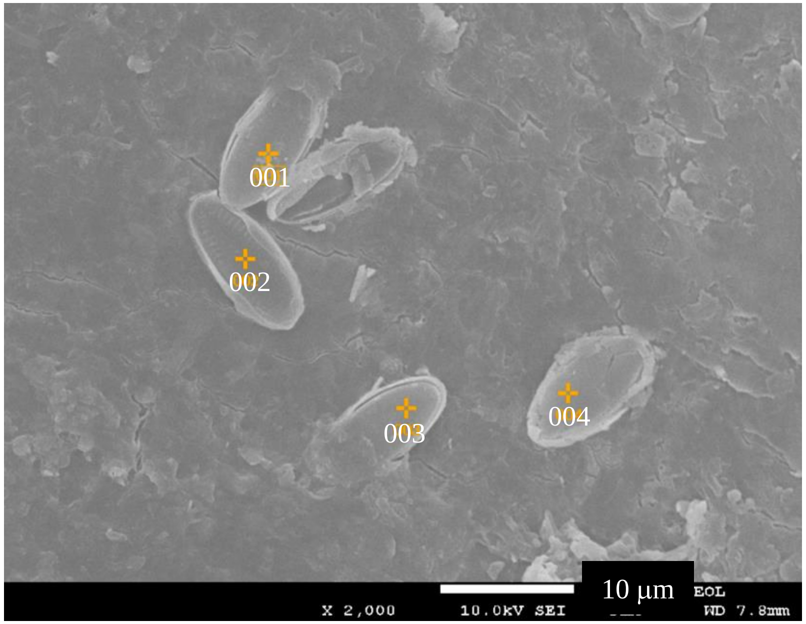

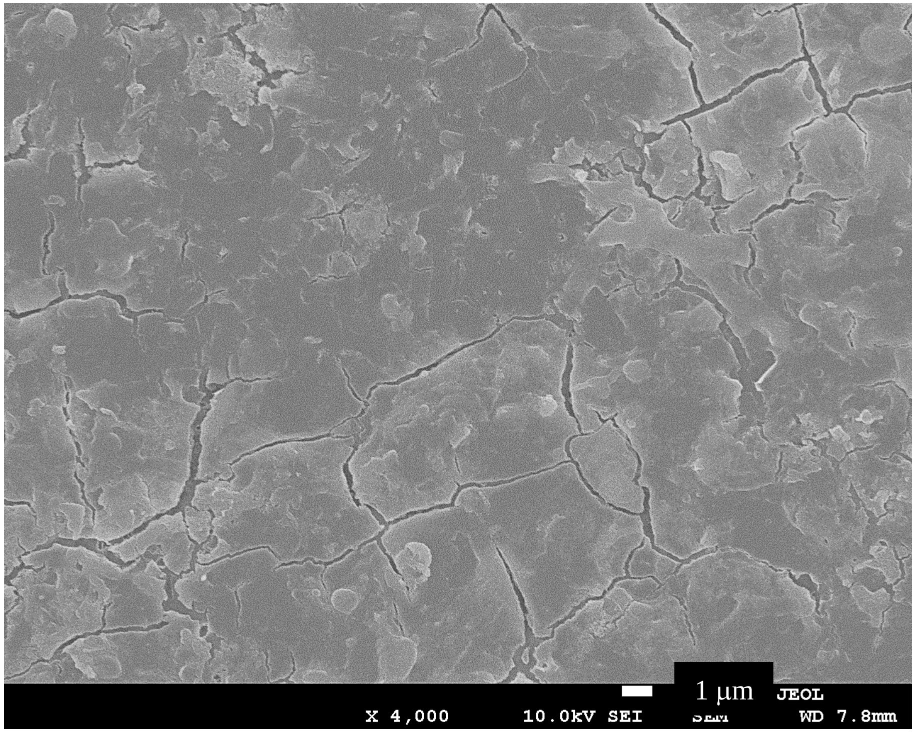

Figure S5 EDX data and SEM images of LLDPE/NPQ sample after 12 months bio-blister degradation test in fresh water (Amazon River Giants) tank at East Idaho Aquarium.

Tank condition information

Tank name	Temp (°C)	Salinity (Specific gravity)	pH
Shark Tank	25.7	1.019	7.9
Amazon(River Giants)	22.7	1.000	7.3

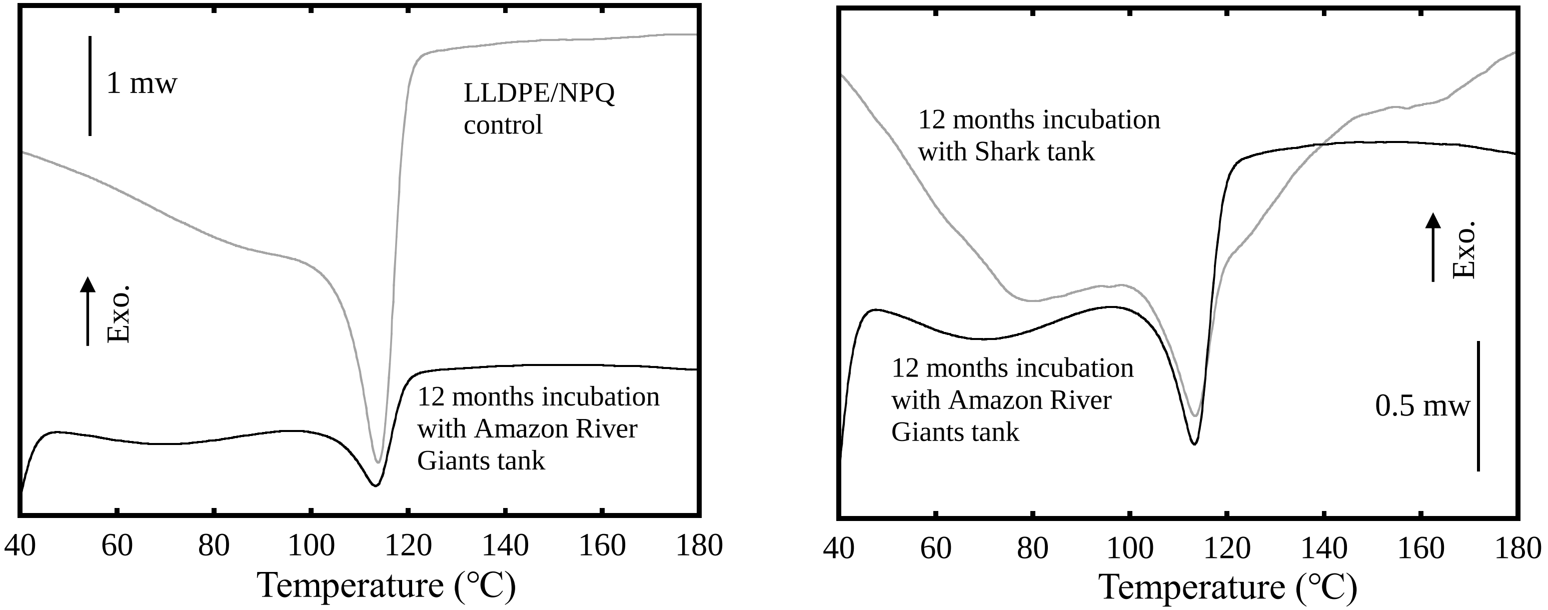

Figure S6 Shark and River Giants tank condition information and comparisons of DSC curves among LLDPE/NPQ samples.

Table S1 Molecular weight information

LLDPE/NPQ sample name	Mn ($\times 10^4$)	Mw ($\times 10^4$)	Mz ($\times 10^4$)	Mw/Mn
Control	3.1	8.7	17	2.9
12 months incubation with Shark tank	3.1	8.3	16	2.7
15 days blister degradation with $K_2S_2O_8$ seawater solution.	0.76	2.7	6.4	3.5

Mn: Number average molecular weight. Mw: Weight average molecular weight.
Mz: Z-average molecular weight.
